# A deep learning model for classification of chondroid tumors on CT images

**DOI:** 10.1186/s12885-025-13951-1

**Published:** 2025-03-28

**Authors:** Felix G. Gassert, Daniel Lang, Nina Hesse, Hans Roland Dürr, Alexander Klein, Luca Kohll, Florian Hinterwimmer, Johanna Luitjens, Stefan Weissinger, Jan C. Peeken, Carolin Mogler, Carolin Knebel, Stefan Bartzsch, Florian T. Gassert, Alexandra S. Gersing

**Affiliations:** 1 Department of Radiology and Biomedical Imaging, University of California San Francisco, 505 Parnassus Ave, San Francisco, CA 94143 USA; 2 Department of Radiology, Klinikum Rechts der Isar, School of Medicine and Health, Technical University of Munich, Ismaninger Strasse 22, 81675 Munich, Germany; 3 Institute of Radiation Medicine, Helmholtz Zentrum München, Ingolstädter Landstraße 1, 85764 Neuherberg, Germany; 4 Department of Radiation Oncology, School of Medicine and Health, Klinikum Rechts der Isar, Technical University of Munich, Ismaninger Strasse 22, 81675 Munich, Germany; 5 Orthopaedic Oncology, Department of Orthopaedics and Trauma Surgery, Musculoskeletal University Center Munich (MUM), LMU Klinikum, University Hospital, LMU Munich, Marchioninistraße 15, 81377 Munich, Germany; 6 Department of Orthopaedics and Sports Orthopaedics, School of Medicine and Health, TUM University Hospital, Technical University of Munich, Ismaninger Strasse 22, 81675 Munich, Germany; 7 Department of Radiology, LMU University Hospital, LMU Munich, Marchioninistraße 13, 80337 Munich, Germany; 8 Department of Pathology, Klinikum Rechts der Isar, School of Medicine and Health, Technical University of Munich, Ismaninger Strasse 22, 81675 Munich, Germany; 9 Department of Neuroradiology, LMU University Hospital, LMU Munich, Marchioninistraße 13, 80337 Munich, Germany; 10 Institute for AI and Informatics in Medicine, School of Medicine and Health, TUM University Hospital, Technical University of Munich, Munich, Germany

**Keywords:** Enchondroma, Chondrosarcoma, Deep learning, Computed tomography

## Abstract

**Background:**

Differentiating chondroid tumors is crucial for proper patient management. This study aimed to develop a deep learning model (DLM) for classifying enchondromas, atypical cartilaginous tumors (ACT), and high-grade chondrosarcomas using CT images.

**Methods:**

This retrospective study analyzed chondroid tumors from two independent cohorts. Tumors were segmented on CT images. A 2D convolutional neural network was developed and tested using split-sample and geographical validation. Four radiologists blinded to patient data and the DLM results with various levels of experience performed readings of the external test dataset for comparison. Performance metrics included accuracy, sensitivity, specificity, and area under the curve (AUC).

**Results:**

CTs from 344 patients (175 women; age = 50.3 ± 14.3 years;) with diagnosed enchondroma (*n* = 124), ACT (*n* = 92) or high-grade chondrosarcoma (*n* = 128) were analyzed. The DLM demonstrated comparable performance to radiologists (*p* > 0.05), achieving an AUC of 0.88 for distinguishing enchondromas from chondrosarcomas and 0.82 for differentiating enchondromas from ACTs. The DLM and musculoskeletal expert showed similar performance in differentiating ACTs from high-grade chondrosarcomas (*p* = 0.26), with an AUC of 0.64 and 0.56, respectively.

**Conclusions:**

The DLM reliably differentiates benign from malignant cartilaginous tumors and is particularly useful for the differentiation between ACTs and Enchondromas, which is challenging based on CT images only. However, the differentiation between ACTs and high-grade chondrosarcomas remains difficult, reflecting known diagnostic challenges in radiology.

## Background

Cartilaginous bone tumors, characterized by tumor cells that produce a chondroid matrix, are divided into benign enchondromas and malignant chondrosarcomas. Chondrosarcomas, the most common primary malignant bone tumors, are classified into various histological grades and subtypes [1, 2]. According to the World Health Organization classification system, Chondrosarcomas grade 1, located in the appendicular skeleton, are now termed “atypical cartilaginous tumors” (ACT) [3]. While enchondromas usually do not require any treatment, ACTs and chondrosarcomas necessitate curettage and/or wide resection [4]. Accurate classification of these tumors is therefore crucial for appropriate treatment.

The final diagnosis of cartilaginous tumors is based on a combination of clinical findings, histopathological results, and imaging studies, and is ideally performed at centers specialized in the treatment of musculoskeletal tumors [5, 6]. While biopsy remains essential in the diagnostic pathway of malignant cartilaginous tumors, the final diagnosis often relies on consensus reached through interdisciplinary tumor board discussions, integrating clinical history, histopathological findings, and radiological assessments [7, 8].

Several studies have investigated imaging parameters to improve the differentiation, especially between ACTs and enchondromas [9–11]. Murphey et al. showed that cortical destruction, periosteal reaction and endosteal scalloping (> 2/3 of cortical thickness) significantly increased the likelihood of an ACT compared to an enchondroma [12]. Nevertheless, radiological differentiation between enchondromas and ACTs remains challenging and highly variable depending on the radiologist’s expertise.

To improve diagnostic accuracy, a deep learning-based approach capable of precisely categorizing cartilaginous tumors as benign or malignant based on CT images only, may be useful for clinical routine. Recently, studies have shown that deep learning models (DLM) reliably assess and detect a variety of musculoskeletal diseases based on medical imaging data [13–15].

More recent studies have extended these findings by incorporating multimodal imaging approaches, such as combining X-ray, CT, and MRI, to enhance classification performance [16]. Additionally, radiomics-based machine learning models have demonstrated promising results in distinguishing enchondromas from atypical cartilaginous tumors (ACTs) and low-grade chondrosarcomas [17, 18].

Therefore, the aim of this study was to develop a DLM specifically designed to differentiate between enchondromas, ACTs, and high-grade chondrosarcomas based on CT images.

## Methods

Approval of the Institutional Review Boards had been obtained prior to this study (Institutional Review Board of the Technical University of Munich, approval number 393/20, and the Ludwig-Maximilian University of Munich, approval number 21–0282). Written informed consent was waived for this retrospective analysis of routinely acquired imaging and clinical data. All analyses are in line with the declaration of Helsinki.

### Data sets

In this study we evaluated cartilaginous tumors on CT images of patients from two university hospitals (Technical University of Munich and the Ludwig-Maximilian University of Munich) treated between 2011 and 2020. These patients had a final diagnosis of either an enchondroma, an ACT or a high-grade chondrosarcoma and preoperative CT images available in the respective picture archiving and communication systems (PACS). The diagnoses were determined based on the consensus of the respective local interdisciplinary tumor board consisting of specialized pathologists, radiologists, and orthopedic tumor surgeons. Patients with enchondroma, ACT or high-grade chondrosarcoma of the hand or foot were excluded from this study. The internal dataset from the Technical University of Munich included 244 patients, divided using split-sample validation into training/validation/hold-out testing sets of 60% (*n* = 146)/ 20% (*n* = 49)/ 20% (*n* = 49). The external test set using geographical validation was obtained from Ludwig-Maximilian University for further, independent testing with a total of 100 (29.07% of the entire dataset) patients. In total, 344 CT-imaging datasets were extracted of patients diagnosed with enchondromas (*n* = 124), ACTs (*n* = 92), and high-grade chondrosarcomas (*n* = 128).

For comparison of diagnostic performance between radiologists and DLM, two radiologic residents with 4 years of experience, one musculoskeletal fellow and one musculoskeletal attending radiologist with 10 and 30 years of experience, respectively, classified the tumors of the external dataset.

**Fig. 1:**
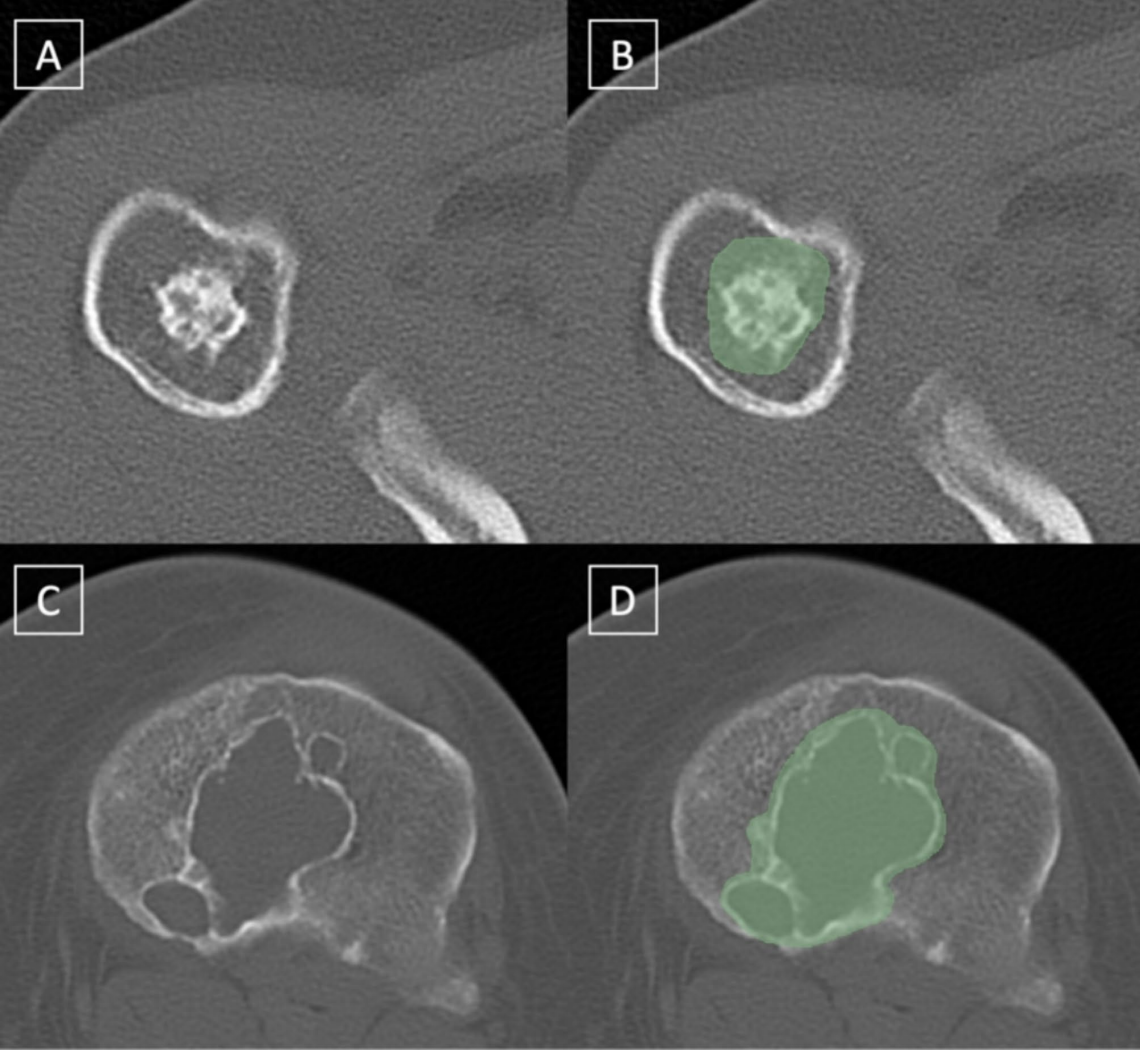
Axial CT Images of patients with an enchondroma (**A**, **B**) and a high-grade chondrosarcoma (G2) (**C**, **D**) with (**B**, **D**) and without the respective segmentations (**A**, **C**)

Portable Network Graphics-files were extracted from Digital Imaging and Communications in Medicine files for further image processing. For the training dataset, segmentations of the tumors were performed by L.K. and F.G.G. and reviewed by a musculoskeletal fellowship trained radiologist (A.S.G) using the open-source software 3D Slicer (version 4.7; www.slicer.org). The radiologists were blinded to the histopathological and clinical data. Segmentations were performed using the entire datasets and all slices on which the tumor was depicted. Figure 1 shows exemplary images of the segmentations of an enchondroma and an ACT. The image containing the largest tumor area was selected, followed by image cropping around the tumor based on the segmentation mask provided by expert radiologists. Thus, a 2D model was designed for image analysis.

### Model architecture and model training

A 2D convolutional neural network (CNN) was trained for classification. For hyperparameter selection, an empirical grid search has been performed, with each model being trained for 100 epochs. The final best model consisted of three convolutional layers of size 16, 32 and 64, each followed by a max pooling layer with a stride of 2 × 2, ending in a fully connected layer of size 128 followed by a single output neuron. Furthermore, an optimal learning rate of 0.001 and a batch size of 32 have been identified. Convolutional and pooling layers featured a kernel size of 3 × 3. Labels of 0.0, 0.5 and 1.0 were assigned to the three classes, reflecting the increase in severity. The mean squared error loss was used for optimization.

### Statistical analysis

Calculations of model metrics and model evaluations were performed using Stata version 18.0. The performance of the models was evaluated with the area under the curve (AUC) obtained from the receiver operating characteristic (ROC) analysis on both the validation set and the independent hold-out test set. Furthermore, final model performance was evaluated using accuracy, sensitivity, specificity and AUC on the independent test-set as defined previously [19]. For performance comparison McNeil’s test was used, and statistical significance was defined as *p* < 0.05. Model training and evaluation was performed by D.L. (5 years’ experience in data analysis) and S.B. (12 years’ experience in data analysis). The intra- and interreader agreement of CT readings was assessed with Fleiss’ κ. To assess the intrareader reliability of the tumor segmentations, ten patients were randomly selected from the internal data set. One radiologist (L.K.) repeated the segmentations three months after the initial segmentation, blinded to the previous results. Segmentations were compared using the dice score.

## Results

### Patient characteristics and data sets

This study included 344 patients (mean age 50.3 ± 14.3 years; 175 women). There were no significant differences in age (*p* = 0.33) or sex (*p* = 0.36) between the internal and external datasets. Based on the consensus of the interdisciplinary tumor board, the internal dataset comprised 108 patients (44%) with enchondromas, 58 patients (24%) with ACTs, and 78 patients (32%) with high-grade chondrosarcomas. The external dataset included 16 patients (16%) with enchondromas, 35 patients (35%) with ACTs, and 49 patients (49%) with high-grade chondrosarcomas. Patient characteristics are summarized in Table 1.

In total, 101 tumors (29.4%) were located in the upper extremity, 130 tumors (37.8%) in the lower extremity, 62 (18%) in the pelvis, and 61 (17.7%) in the remaining trunk.

**Table 1:** Patient characteristics

	Internal dataset (*n* = 244)	External dataset (*n* = 100)	All (*n* = 344)	*p*-value
Sex				
Women	128	47	175	0.36
Men	116	53	169	
Age [years]	49.8 ± 14.6	51.5 ± 15.3	50.3 ± 14.8	0.33
Grading				
Enchondroma	108	16	124	< 0.001
ACT	58	34	92	
HGCS	78	50	128	

ACT = atypical cartilaginous tumor; HGCS = High-grade Chondrosarcomas

### Performance of the DLM and the radiologists on the external test set

For comparison of diagnostic performance, the DLM as well as the four radiologists performed an analysis of the external test set. All results are summarized in Table 2.

**Table 2:** Performance parameters of the four readers and the deep learning algorithm for the differentiation between enchondromas vs. chondrosarcomas (G1-G3), enchondromas vs. acts as well as acts vs. High-Grade chondrosarcomas on the external dataset

Enchondromas vs. Chondrosarcomas (G1 - G3)					
	Resident 1	Resident 2	Fellowship	Expert	Algorithm
Accuracy	0.770	0.840	0.870	0.900	0.750
Positive Predictive Value	0.772	0.847	0.844	0.889	0.736
Negative Predictive Value	0.762	0.821	0.957	0.929	0.846
Sensitivity	0.924	0.924	0.985	0.970	0.970
Specificity	0.471	0.676	0.647	0.765	0.324
Balanced Accuracy	0.697	0.800	0.816	0.867	0.647
F1-Score	0.841	0.884	0.909	0.928	0.837
AUC	0.810	0.891	0.910	0.932	0.884

Enchondromas vs. ACTs					
	Resident 1	Resident 2	Fellowship	Expert	Algorithm
Accuracy	0.667	0.772	0.772	0.842	0.561
Positive Predictive Value	0.550	0.656	0.647	0.733	0.477
Negative Predictive Value	0.941	0.920	0.957	0.963	0.846
Sensitivity	0.957	0.913	0.957	0.957	0.913
Specificity	0.471	0.676	0.647	0.765	0.324
Balanced Accuracy	0.714	0.795	0.802	0.861	0.618
F1-Score	0.698	0.764	0.772	0.830	0.627
AUC	0.812	0.872	0.868	0.919	0.816

ACT vs. High-Grad Chondrosarcoma					
	Resident 1	Resident 2	Fellowship	Expert	Algorithm
Accuracy	0.545	0.545	0.727	0.652	0.561
Positive Predictive Value	0.686	0.686	0.719	0.685	0.719
Negative Predictive Value	0.387	0.387	0.778	0.500	0.412
Sensitivity	0.558	0.558	0.953	0.860	0.535
Specificity	0.522	0.522	0.304	0.261	0.609
Balanced Accuracy	0.540	0.540	0.629	0.561	0.572
F1-Score	0.615	0.615	0.820	0.763	0.613
AUC	0.525	0.541	0.630	0.561	0.637

*Reader 1 and 2 = residents; Reader 3 = fellowship trained radiologist; Reader 4 = musculoskeletal attending radiologist; AUC = area under the receiver operating characteristic. curve*

#### Differentiation enchondromas vs. chondrosarcomas (G1 - G3)

**Fig. 2:**
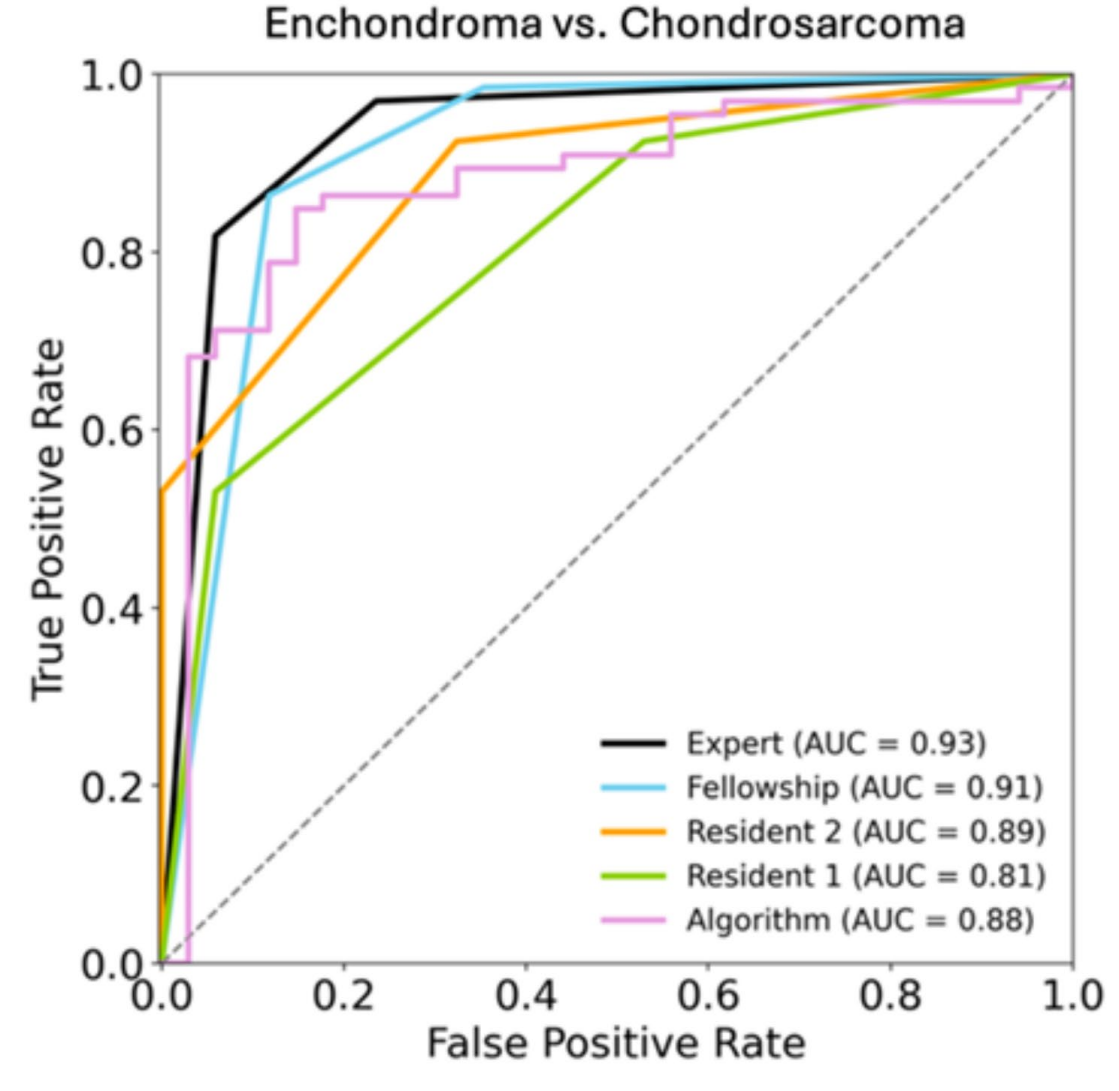
ROC-Analysis of the radiologists and the algorithm for the differentiation of enchondromas and chondrosarcomas (G1-G3). Reader 1 and 2 = residents; Reader 3 = fellowship trained radiologist; Reader 4 = musculoskeletal attending radiologist; ROC = receiver operating characteristic; AUC = area under the curve

The DLM achieved a sensitivity, specificity, and accuracy of 97.0%, 32.4%, and 75.0% with an AUC of 0.88 for the differentiation between benign and malignant cartilaginous tumors on CT images. The overall sensitivity, specificity, accuracy of the residents’ readings were 92.4%, 47.1%, and 77.0%, and 92.4%, 67.6%, and 84.0%, respectively, with AUC values of 0.81 and 0.89. For the fellowship trained radiologist and the musculoskeletal attending radiologist, the overall sensitivity, specificity, accuracy were 98.5%, 64.7%, and 87.0%, and 97.0%, 76.5%, and 90.0%, respectively, with AUC values of 0.91 and 0.93. There was no significant difference found between the performance of the DLM and the musculoskeletal fellowship-trained radiologist (*p* = 0.19). Figure 2 shows the ROC analysis for the differentiation between benign and malignant chondroid tumors.

#### Differentiation enchondromas vs. ACTs

**Fig. 3:**
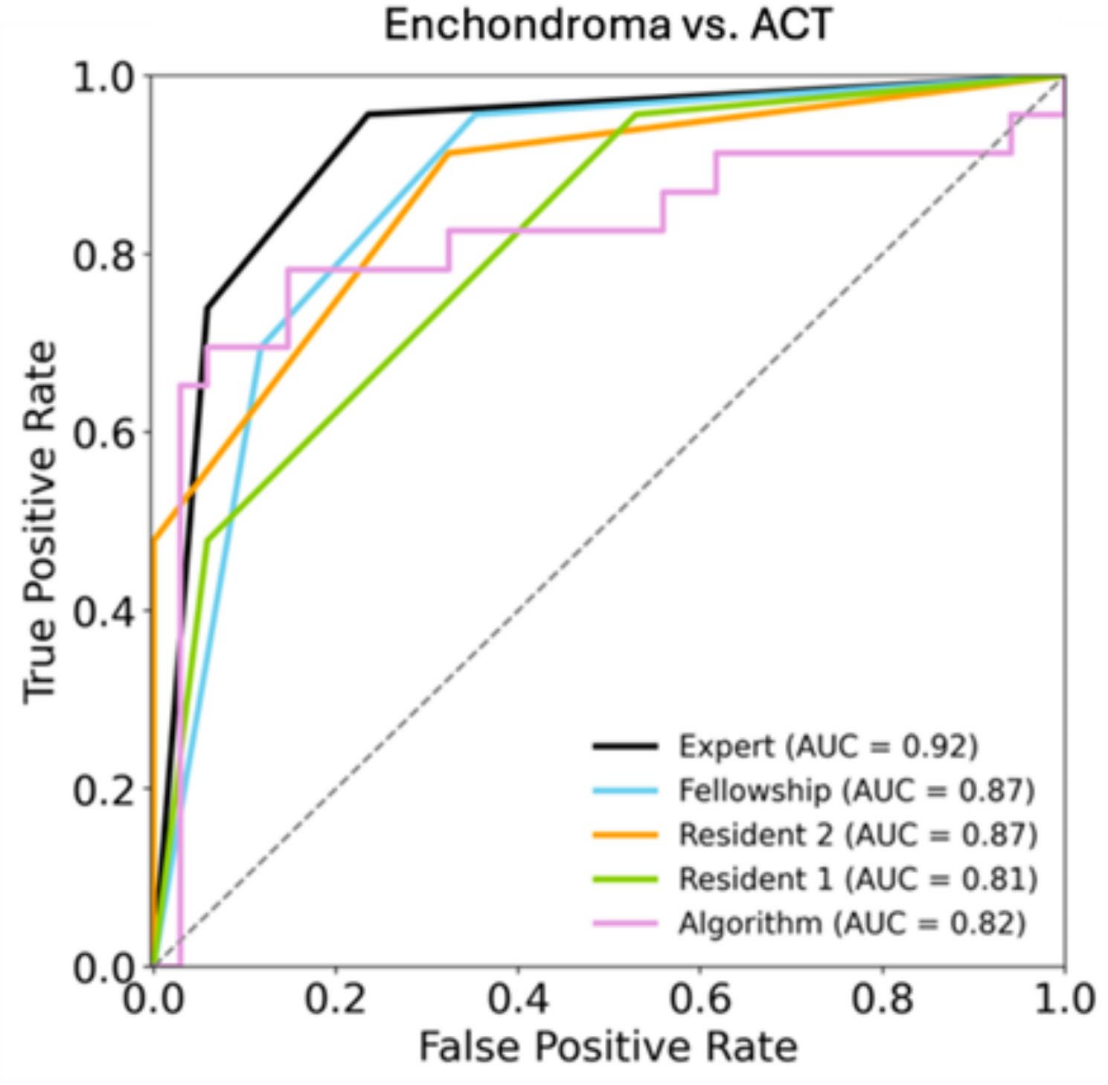
ROC-Analysis of the radiologists and the algorithm for the differentiation of enchondromas and atypical chondroid tumors. Reader 1 and 2 = residents; Reader 3 = fellowship trained radiologist; Reader 4 = musculoskeletal attending radiologist; ROC = receiver operating characteristic; AUC = area under the curve

For the differentiation between enchondromas and ACTs the DLM achieved an overall sensitivity, specificity, and accuracy of 91.3%, 32.4%, and 56.1% with an AUC of 0.82. The overall sensitivity, specificity, and accuracy of the residents’ readings was 95.7%, 47.1%, and 66.7%, and 91.3%, 67.6%, and 77.2%, respectively, with AUC values of 0.81 and 0.87. The fellowship trained radiologist and the musculoskeletal attending radiologist achieved an overall sensitivity, specificity, and accuracy of 95.7%, 64.7%, and 77.2%, and 95.7%, 76.5%, and 84.2%, respectively, with AUC values of 0.87 and 0.92. There was no significant difference between the performance of the DLM and the four radiologic readers (*p* = 0.96, 0.33, 0.41, and 0.82). Figure 3 shows the ROC analysis for the differentiation between enchondromas and ACTs.

#### Differentiation ACTs vs. high-grade chondrosarcomas

**Fig. 4:**
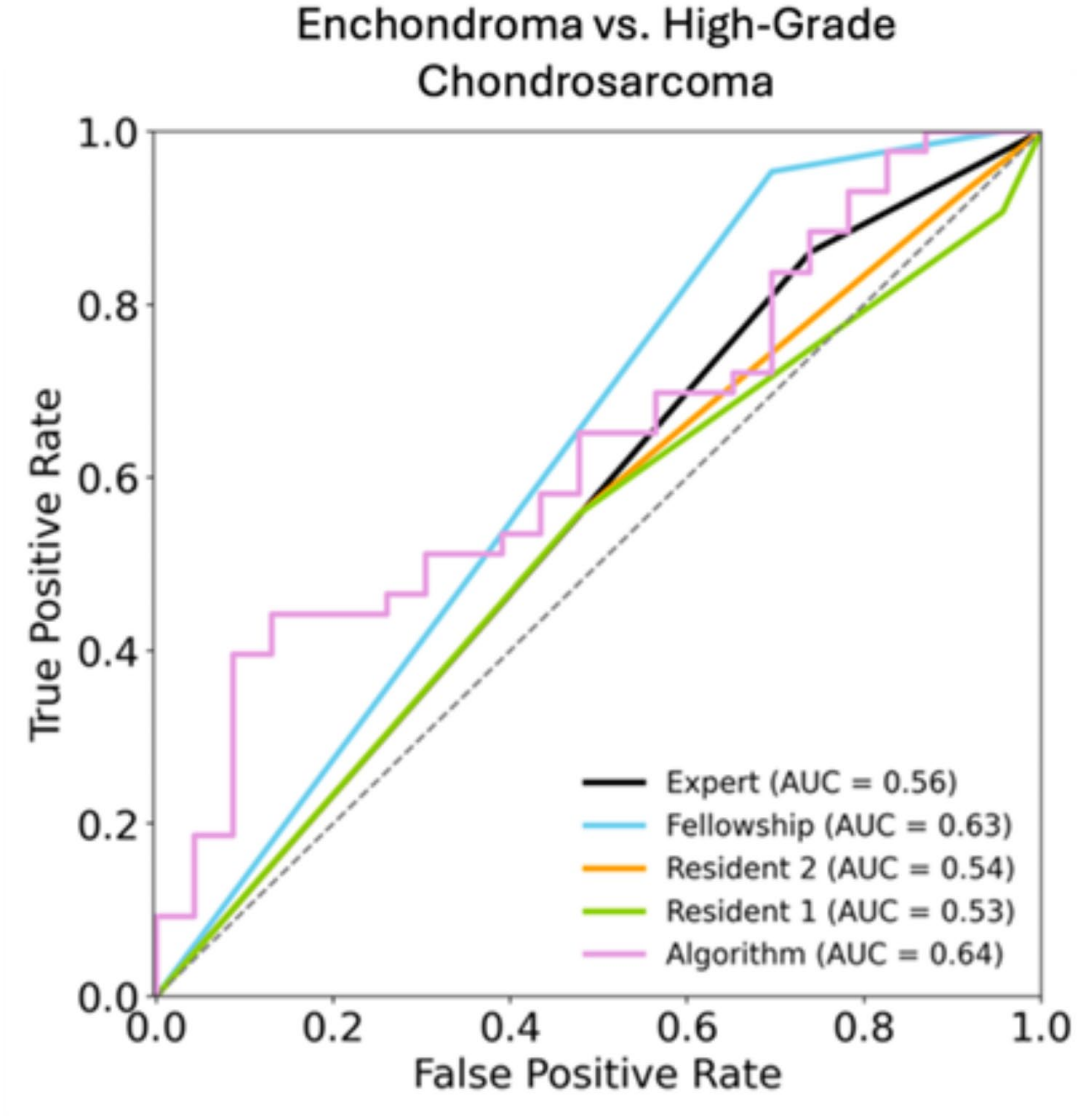
ROC-Analysis of the radiologists and the algorithm for the differentiation of atypical chondroid tumors and high-grade chondrosarcomas. Reader 1 and 2 = residents; Reader 3 = fellowship trained radiologist; Reader 4 = musculoskeletal attending radiologist; ROC = receiver operating characteristic; AUC = area under the ROC curve

Regarding the differentiation between ACTs and high-grade chondrosarcomas the DLM achieved a sensitivity, specificity, and accuracy of 53.5%, 60.9%, and 56.1% with an AUC of 0.64. The overall sensitivity, specificity, and accuracy of the residents’ readings were 55.8%, 52.2%, and 54.5%, and 55.8%, 52.2%, and 54.4%, respectively, with an AUC of 0.53 and 0.54. The overall sensitivity, specificity, and accuracy of the fellowship-trained radiologist and the musculoskeletal radiology attending were 95.3%, 30.4%, and 72.7%, and 86.0%, 26.1%, and 65.2%, respectively, with AUCs of 0.63 and 0.56. No significant difference was found between the performance of the DLM and the musculoskeletal attending radiologist (*p* = 0.26). Figure 4 shows the AUC curve for the differentiation between ACTs and enchondromas.

### Inter-observer and intra-observer reliability

The inter-observer reliability was substantial to almost perfect for all criteria (κ = 0.76–1.00) and the intra-observer reliability was excellent (κ = 0.87–0.96), respectively. The dice score for the intrareader reliability was 0.97.

## Discussion

The differentiation of chondroid tumors, especially between enchondromas and ACTs, is challenging on CT images yet crucial for determining appropriate therapeutic treatment and ensuring optimal patient outcomes. ACTs require a curettage as well as clinical and imaging follow-ups, whereas enchondromas typically do not require surgical intervention, unless symptomatic, and less frequent follow-ups. Therefore, this study aimed to develop a DLM for differentiating benign, intermediate and malignant chondroid tumors on CT images, comparing its diagnostic performance with that of radiologists. The DLM demonstrated a comparable performance to the experienced musculoskeletal radiologists.

To our knowledge, this is the first study showing the feasibility of using a DLM for the differentiation of chondroid tumors. In the past several studies have evaluated the diagnostic value of different imaging features for the differentiation of chondroid tumors [9–11]. Murphey et al. showed that cortical destruction, periosteal reaction, and endosteal scalloping (> 2/3 of cortical thickness) significantly increase the likelihood of a chondrosarcoma compared to an enchondroma [12]. Additionally, Crim et al. and Douis et al. identified tumor size, cortical breakthrough, periosteal reaction and matrix calcifications as key differentiating criteria [9, 10]. Nevertheless, differentiating enchondromas from ACTs radiologically remains challenging and varies with the radiologist’s expertise.

In our study, we used CT images for development of the DLM, although MRI can provide additional information. A recent study demonstrated that both CT and MRI show suggestive signs which can help to adequately differentiate enchondromas from ACTs in long bones. However, these features are rare, and a combination of CT and MRI features does not substantially improve the diagnostic performance [20].

Previous studies have shown the feasibility of using DLMs to differentiate between benign and malignant musculoskeletal tumors across various imaging modalities. Von Schacky et al., Liu at al., and He et al. developed CNN-based DLMs for the classification of benign, malignant, and intermediate primary bone tumors on radiographs in cohorts of 934, 643, and 1536 patients, respectively, achieving an AUC of up to 0.916 [15, 21, 22]. Several deep learning methods have been developed for detecting and classifying bone tumors using CT and MR images. For instance, a DLM based on radiomics in CT images and clinical parameters for discriminating between benign and malignant sacral tumors in 459 patients achieved an AUC of 0.83 [23]. Another DLM by Eweje et al. based on routine MR images and patient demographics achieved for the classification of benign and malignant bone lesions an AUC of 0.79 [24]. The performance of our DLM, with an AUC of 0.88 for differentiating between enchondromas and chondrosarcomas and an AUC of 0.82 for differentiating between enchondromas and ACTs, is comparable to those previous studies [23, 24].

In this study, a 2D CNN was employed, optimized via a mean squared error loss function, to facilitate the differential diagnosis of chondroid tumors utilizing CT data. The performance of the model, as demonstrated by the AUC scores, closely paralleled the diagnostic accuracy of experienced musculoskeletal radiologists. Such comparable efficacy across distinct patient cohorts underscores the potential of the model for generalization, an essential criterion for clinical translatability. The incorporation of this DLM into the diagnostic protocol may offer substantial clinical value, particularly as a supportive tool for radiologists with varying levels of expertise or experts in other fields aside from oncological or bone imaging. It can act as a supplementary computational consultation, thereby streamlining the diagnostic process and potentially impacting patient management strategies. Future research could explore model refinement through the integration of multimodal imaging variables and relevant clinical parameters.

While this study utilized a 2D CNN for tumor classification, future research could explore the potential advantages of 3D CNN architectures. A 3D CNN could leverage volumetric information from CT scans, potentially enhancing feature extraction and improving classification accuracy [25]. Prior studies have demonstrated that 3D deep learning models can be beneficial in various medical imaging tasks, particularly for segmenting and analyzing complex anatomical structures [26]. However, the increased computational cost, need for larger annotated datasets, and potential challenges in clinical interpretability must be considered. Furthermore, current radiological workflows predominantly rely on 2D slice-based assessments, making our approach more aligned with real-world clinical practice. Future studies could evaluate the feasibility of 3D CNNs in this setting and compare their performance against 2D approaches.

In addition to deep learning-based classification, advanced imaging modalities such as SPECT/CT radiomics have also shown potential in differentiating enchondromas from ACTs [27]. By extracting quantitative imaging features from functional imaging data, these approaches may complement conventional CT-based models. Future studies could explore the integration of SPECT/CT-based radiomics with deep learning models to enhance diagnostic accuracy.

One key finding of this study is the notable difference in classification performance between the differentiation of enchondromas from ACTs and high-grade chondrosarcomas, and the differentiation of ACTs from high-grade chondrosarcomas. This reflects a well-known challenge in musculoskeletal radiology, as these tumor types share overlapping imaging features on CT, particularly in terms of matrix mineralization and cortical involvement. Additionally, tumor heterogeneity within ACTs and high-grade chondrosarcomas may contribute to variability in their imaging presentation, further complicating classification. Previous studies have suggested that MRI, particularly contrast-enhanced and diffusion-weighted sequences, may offer additional diagnostic value in differentiating these entities. Future studies could explore multimodal deep learning approaches that integrate MRI data or employ radiomics-based feature extraction to enhance classification performance.

This study has limitations. Firstly, there has been a significant difference in the composition of the internal and external data set with a relatively low number of enchondromas in the external data set. Nevertheless, the overall number of patients in this study, to the authors knowledge, is the highest number of patients published in a single study [9, 10]. Secondly, in this study only patients with biopsy proven lesions were included, which may cause a bias towards more malignant looking lesions on the CT images. And lastly, our study did not include clinical parameters due to the retrospective design and large time frame of this study which was necessary to achieve such a big data set. Future studies may include clinical data as well as a combination of CT and MR images. And lastly, one of the main challenges in this study was the differentiation between ACTs and high-grade chondrosarcomas, where the model demonstrated lower performance compared to other classifications. This is consistent with prior studies reporting that even experienced musculoskeletal radiologists struggle with this distinction based on CT images alone. The overlapping imaging characteristics of ACTs and high-grade chondrosarcomas contribute to this difficulty. Future studies could explore the integration of MRI features or clinical parameters to enhance diagnostic accuracy.

## Conclusions

In conclusion, the DLM reliably differentiates benign, intermediate, and malignant cartilaginous tumors, particularly enchondromas and ACTs, which are often difficult to distinguish based on CT imaging alone. However, its performance in differentiating ACTs from high-grade chondrosarcomas was more limited, in line with known diagnostic challenges.

## Data Availability

The datasets used and/or analysed during the current study are available from the corresponding author on reasonable request.

## Abbreviations

ACT: Atypical cartilaginous tumors
AUC: Area under the curve
CNN: Convolutional neural network
DLM: Deep learning model
PACS: Picture archiving and communication systems
ROC: Receiver operating characteristic
